# Hashimoto Thyroiditis in a Patient With Dubowitz Syndrome and Short Stature

**DOI:** 10.7759/cureus.48440

**Published:** 2023-11-07

**Authors:** Hadil Basma, Sara Dbouk, Zeinab Issa

**Affiliations:** 1 Endocrinology Department, Lebanese University Faculty of Medicine, Beirut, LBN; 2 Endocrinology and Diabetes, Bahman Hospital, Beirut, LBN

**Keywords:** hashimoto thyroiditis, short stature, growth hormone, mental disability, dubowitz syndrome

## Abstract

Dubowitz syndrome (DS) is a rare genetic disorder characterized by multiple morphological abnormalities, short stature, and different degrees of mental disability. Endocrinological evaluation should be done for these subjects, as they can suffer from multiple hormonal derangements. We present a case of a 12-year-old Lebanese girl, diagnosed with Dubowitz syndrome, who presented to our clinic for short stature. She had received growth hormones (GHs) and improved her height. More investigations showed the presence of Hashimoto thyroiditis with normal thyroid stimulating hormone, so hormonal follow-up was recommended. The association between Dubowitz syndrome and Hashimoto thyroiditis has not been described so far. Thus, in the setting of this syndrome, it is worthwhile to check for growth hormone deficiency and Hashimoto's thyroiditis.

## Introduction

Dubowitz syndrome (DS) is a rare autosomal recessive disorder characterized by some morphological features such as facial abnormalities, microcephaly, and short stature, with different degrees of intellectual disability ranging from mild to severe. Patients with this disorder suffer from deficits in multiple skills, including speech and language, reasoning, memory, and self-help, as well as abnormal psychomotor functions. It is suggested that it’s of genetic etiology [1]. Multiple genes, such as SKIV2L, SLC35C1, BRCA1, and NSUN2, are involved in the pathogenesis of this syndrome. Subjects with DS are at higher risk of malignancy and hematological disorders. Literature reports over 200 individuals with Dubowitz or a "Dubowitz-like" condition [2]. The diagnosis is established by the clinical and facial characteristics.

Patients with DS suffer from growth impairment during the prenatal and postnatal periods. However, the cause of the growth failure is still undetermined [3].

Subjects with a Dubowitz phenotype should be investigated for endocrinological anomalies to recognize potentially life-threatening conditions such as adrenal insufficiency, as reported in one case [3]. So far, there is no known association between Dubowitz syndrome and Hashimoto thyroiditis and no reported cases.

Our case is a young girl with known DS who was found to have growth impairment and Hashimoto thyroiditis.

## Case presentation

We present a case of a 12-year-old Lebanese girl diagnosed with Dubowitz syndrome who presented to our clinic for short stature. She has two sisters with normal development and no consanguinity between her parents. There is no significant family history. She was born by C-section delivery on term, and no neonatal intensive care was needed. Her mother mentioned a normal pregnancy course and denied the use of any medication, smoking, or alcohol consumption before or during pregnancy. Between two and four years of age, she was found to be an overactive girl, with developmental retardation and limited skills compared to her age level. She had retarded speech with respect to her siblings and had mildly delayed walking, so in general, her milestones were delayed with respect to her age-matched children. Her vaccines were received as recommended. Morphologically, she had abnormal facial features. After many investigations, she was diagnosed with Dubowitz syndrome.

The patient was found to have short stature at the age of 2 years; karyotype was done and was normal for copy and number of chromosomes 13, 18, 21, and X. DNA analysis using the Bacs-on-Beads Assay excluded the presence of any micro-deletions or micro-duplications. Magnetic resonance imaging (MRI) of the brain was normal. An X-ray to determine the bone age was done, and it was within two standard deviations of the chronological age. Insulin growth factor 1 (IGF1) and IGF1 binding protein were normal (Table 1). Laboratory tests done at the first presentation (at two years) are shown in Table 1.

**Table 1:** Laboratory tests at first presentation for short stature. TSH: thyroid-stimulating hormone, FT4: free thyroxin 4, IGF1: insulin growth factor 1.

Test	Unit	Patient’s test value	Normal range for her age
White blood cells	/cu.mm	4800	3.8–10.4
Polymorphonuclears	%	46	40–60
Lymphocytes	%	43	20–40
Eosinophils	%	2	1–4
Basophils	%	1	0.5–1
Monocytes	%	8	2–8
Hemoglobin	g/dl	13.3	11.9–14.8
Hematocrit	%	39	35–43
Mean corpuscular volume	Femtoliter	89	82.5–98
Platelets	/cu.mm	185,000	153,000–361,000
Creatinine	mg/dl	0.5	0.5–1
TSH	mIU/L	1.92	0.3–5
FT4	ng/dl	1.32	0.8–2.3
IGF1	ng/ml	119	38–230
IGF1-binding protein	ng/ml	3450	1872–5373

At the age of two years, her height was 79 cm and her weight was 10 kg (on the fifth percentile for both), noting that her mother’s and father’s heights are 160 and 170 cm, respectively, so she lies below the midparental height.

**Figure 1:**
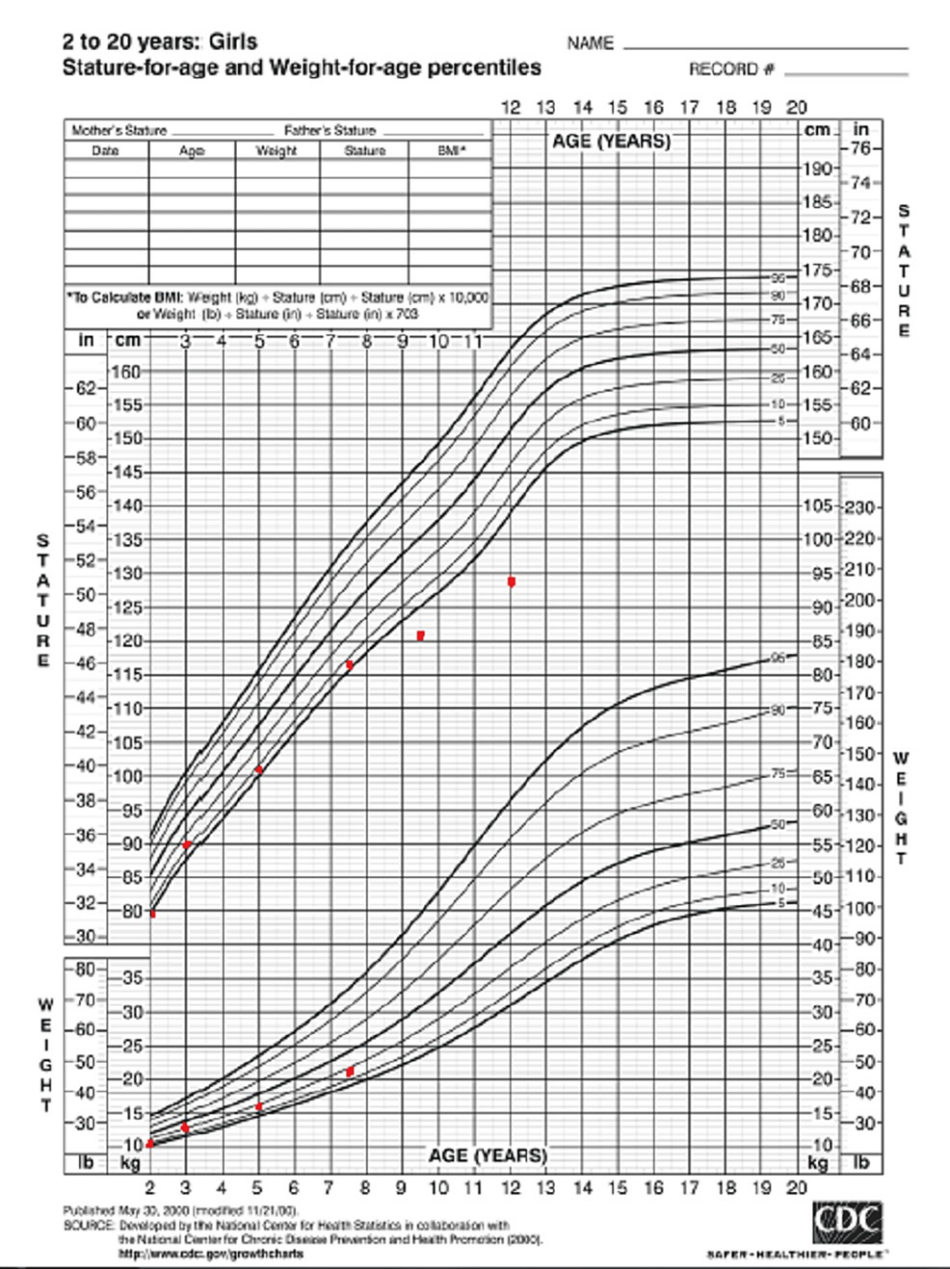
Patient’s height and weight during growth. This figure represents the growth chart of the patient, where the red dots correspond to the height and weight of the patient over the years.

Despite the normal laboratory tests, she was started on growth hormone (GH) treatment for short stature. Her height increased to the 10th percentile at the age of three years after starting treatment with growth hormone at a dose of 0.03 mg per kg of body weight per day and progressed adequately on the 10th percentile line until the age of seven years and six months when the drug was suddenly stopped, and her height dropped to less than the 5th percentile at the age of nine years and six months and stayed under the curve till the age of 12 years when she presented to our endocrinology clinic. The progression of her height and weight over the years is shown in Table 2 and Figure 1.

**Table 2:** Patient’s height and weight during growth.

Age (years)	Height (cm)	Weight (kg)
2	79	10
3	90	12.5
5	101	16
7.5	117	22
9.5	121	-
12	129	-

Upon presentation to our clinic at the age of 12 years, her physical exam showed no enlargement of the thyroid gland and no palpable nodules. Some investigations were done, and the results are as follows in Table 3.

**Table 3:** Laboratory tests at 12 years old. FSH: follicle-stimulating hormone, LH: luteinizing hormone, TSH: thyroid-stimulating hormone, IGF1: insulin growth factor 1, SGPT: serum glutamate pyruvate transaminase.

Test	Unit	Patient's test value	Normal range for her age
Estradiol	pg/ml	5	<20
FSH	mIU/ml	1.38	0–4
LH	IU/L	0.19	0.03–3.9
TSH	mIU/L	3.28	0.53–5.16
Anti-thyroid peroxidase	IU/ml	84	<9
Anti-thyroglobulin	IU/ml	231.6	<116
IGF1	ng/ml	69.1	158–375
Calcium	mg/dl	9.35	8.5–10.2
SGPT	µ/l	14	7–56

According to the results, the patient has positive auto-antibodies for Hashimoto thyroiditis (anti-thyroid peroxidase and anti-thyroglobulin) and low IGF1. At this time, she was restarted on growth hormone replacement.

## Discussion

Dubowitz syndrome is a rare genetic disorder characterized by multiple congenital defects, growth retardation, intellectual incompetence, and other multisystemic features [4]. Microcephaly, short stature, and low body weight are common features of patients with DS. Physical development is often delayed months to years from the actual age of the child [1].

GH deficiency may be a feature of DS. It requires a brain MRI with repeated measurements of IGF-1 because GH deficiency may be transitory [3]. In our case, the first IGF1 and MRI brains were normal. The repeated IGF-1 level after many years was low, and since she had benefited from GH analog therapy, she was restarted on treatment.

The origin of growth impairment is still unknown, but there are multiple proposed mechanisms: GH deficiency, gene alterations involving the GH-IGF1 axis, and derangement in specific brain structures during fetal development [3].

Regarding hypothyroidism, our patient was diagnosed with Hashimoto thyroiditis, which is a chronic autoimmune thyroid disease attributed to two elements that have to be fully understood: genetic factors and environmental conditions [5]. The diagnosis was based on her autoimmune profile with positive antibodies. She was not given treatment since her hormonal workup was normal.

Our case signals the importance of GH deficiency assessment and its replacement in patients with DS. Also, it urges clinicians to check for autoimmune thyroid disease even if the hormonal workup was normal in order to have a follow-up and assess the need for thyroid hormone replacement in later visits.

## Conclusions

We present a girl diagnosed with DS who attended the endocrinology clinic for short stature. Investigations showed low IGF1 and Hashimoto thyroiditis. The combination of DS and GH deficiency has rarely been reported before. In addition, the association between DS and Hashimoto thyroiditis has not been described so far. Thus, it is worth checking for GH deficiency and Hashimoto thyroiditis in patients diagnosed with DS to receive the proper treatment.
